# Deep RNA sequencing reveals the smallest known mitochondrial micro exon in animals: The placozoan *cox1* single base pair exon

**DOI:** 10.1371/journal.pone.0177959

**Published:** 2017-05-18

**Authors:** Hans-Jürgen Osigus, Michael Eitel, Bernd Schierwater

**Affiliations:** 1 ITZ, Ecology & Evolution, Stiftung Tierärztliche Hochschule Hannover, Hannover, Germany; 2 Department of Ecology and Evolutionary Biology, Yale University, New Haven, Connecticut, United States of America; 3 Sackler Institute for Comparative Genomics and Division of Invertebrate Zoology, American Museum of Natural History, New York, New York, United States of America; International Centre for Genetic Engineering and Biotechnology, ITALY

## Abstract

The phylum Placozoa holds a key position for our understanding of the evolution of mitochondrial genomes in Metazoa. Placozoans possess large mitochondrial genomes which harbor several remarkable characteristics such as a fragmented *cox1* gene and trans-splicing *cox1* introns. A previous study also suggested the existence of *cox1* mRNA editing in *Trichoplax adhaerens*, yet the only formally described species in the phylum Placozoa. We have analyzed RNA-seq data of the undescribed sister species, Placozoa sp. H2 (“Panama” clone), with special focus on the mitochondrial mRNA. While we did not find support for a previously postulated *cox1* mRNA editing mechanism, we surprisingly found two independent transcripts representing intermediate *cox1* mRNA splicing stages. Both transcripts consist of partial *cox1* exon as well as overlapping intron fragments. The data suggest that the *cox1* gene harbors a single base pair (cytosine) micro exon. Furthermore, conserved group I intron structures flank this unique micro exon also in other placozoans. We discuss the evolutionary origin of this micro exon in the context of a self-splicing intron gain in the *cox1* gene of the last common ancestor of extant placozoans.

## Introduction

**Fig 1:**
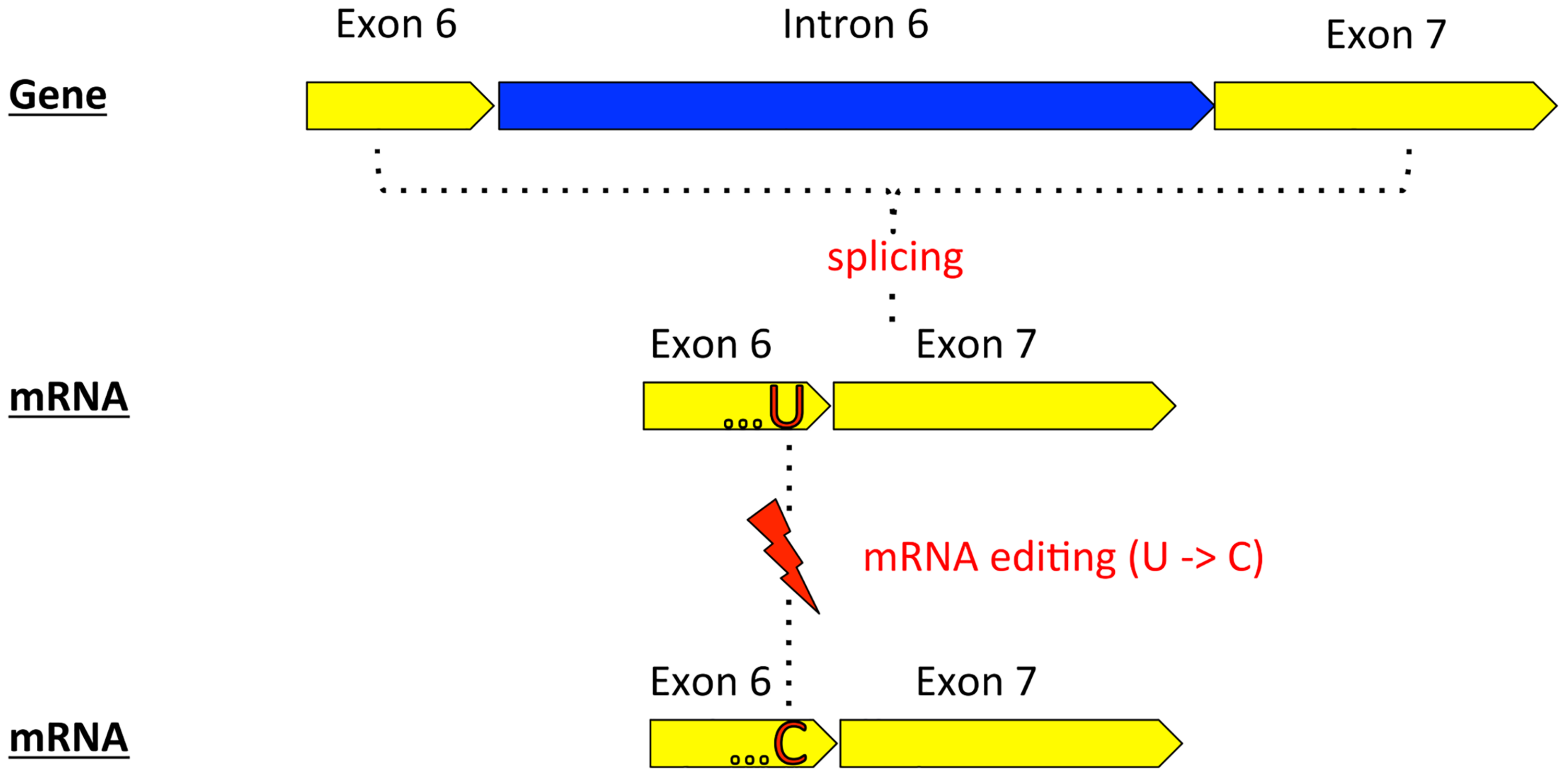
Placozoan *cox1* “mRNA editing” scenario. The shown scenario is based on *Trichoplax adhaerens* EST data (Burger *et al*., 2009). The figure only shows *cox1* exons 6 and 7 and the intron between them (following NC_008151). After splicing of exons the “U” at the 3’ end of exon 6 is converted to a “C” by mRNA editing. Exons and introns are illustrated in yellow and blue, respectively. mRNA editing (“U-to-C”) is illustrated by red lightning. For further explanations see text and Burger *et al*., 2009.

The evolution of mitochondrial (mt) genomes in Metazoa has raised intriguing discussions from several perspectives and refuted the picture of uniform animal mtDNA characteristics (e.g. [1–4]). Exceptional examples from Bilateria include Doubly Uniparental Inheritance (DUI) of mtDNA in molluscs or small circular mtDNA molecules in lice (e.g. [5, 6]). Non-bilaterian animals provide an even more remarkable variety of unusual mtDNA features. The derived Ctenophora (cf. [7]), for instance, possess highly reduced circular mitochondrial genomes [8], while different Porifera and Cnidaria may have linear and sometimes even fragmented mitochondrial genomes [9, 10]. Some of the largest animal mitochondrial genomes are found in the phylum Placozoa [11–13]. As large circular mitochondrial genomes are also found in unicellular organisms (including the choanoflagellate *Monosiga brevicollis* [14]) it has been postulated that placozoan mitochondrial genomes have retained several ancestral characteristics of metazoan mitochondrial genomes [11]. The large mitochondrial genome size, however, is just one of several remarkable mitochondrial genome features. Placozoan mitochondrial genomes show a high tendency for structural changes (i.e. inversions, translocations, insertions or deletions), while sequence evolution rates of protein coding sequences are comparatively low [12]. Another remarkable feature is the variable number of introns in some mitochondrial genes (*cox1*, *nad5* and 16S rDNA) in different placozoans, recommending placozoans as an ideal model system to study the evolution of metazoan mitochondrial introns and mRNA processing or splicing mechanisms. Former analyses of the placozoan *cox1* gene already revealed the presence of several cis-splicing group I and group II introns [11–13]. *Cox1* introns are also found in some Porifera and Cnidaria, but the *cox1* fragmentation in Placozoa is unique among metazoans. To date up to eight exons have been identified in some placozoans. Even more remarkable is the presence of two trans-spliced group IB introns in the *cox1* gene of all placozoans, suggesting that trans-spliced *cox1* introns may already have existed in the last common ancestor of all extant placozoans, and putatively also in the “urmetazoon” (the hypothetical ancestor of the Metazoa) (cf. [15–17]). As another surprise a former analysis of *Trichoplax adhaerens* EST data indicated *cox1* U-to-C mRNA editing in this species ([18], cf. Fig 1) and sparked discussion about the ancestral state of mitochondrial mRNA editing in animals [3]. Although mitochondrial mRNA editing has been found in various other animals (reviewed in [19]) the underlying mechanisms are incompletely understood. The patchy distribution of different mechanisms suggests multiple independent origins. We here re-examine the postulated mRNA editing in the placozoan *cox1* gene in full detail using high-throughput RNA-seq data from Placozoa sp. H2 “Panama”, a sister species to *Trichoplax adhaerens* (Kamm *et al*., in prep.). While we do not find support for U-to-C mRNA editing in our data we do find compelling evidence for a single base pair *cox1* micro exon “C” (cytosine). Ultra short micro exons (from a few nucleotides down to a single) have previously been reported in nuclear as well as mitochondrial genes in other eukaryotes (e.g. [20, 21], and references therein), but this is the first time a single-nucleotide exon has been identified in an animal mitochondrion. Furthermore, functional studies on the regulation of nuclear micro exon splicing as well as on the severe effects of misregulation have been conducted for instance in Bilateria [22, 23]. For the placozoan *cox1* micro exon we here show that this single base pair exon, and not mRNA editing, is indispensable to maintain an evolutionary conserved histidine in the mature *cox1* protein in placozoans.

## Results and discussion

The reconstruction of the complete mitochondrial genome of Placozoa sp. H2 “Panama” revealed a fragmented *cox1* structure identical to the one from *Trichoplax adhaerens* [11, 18]. In detail, the *cox1* gene is fragmented into nine exons (“eight” in previous annotations, see below) encoded on different strands of the circular mitochondrial genome. The unusual 11bp-exon 4, a shared feature in all placozoan *cox1* genes sequenced so far, is also present in the Placozoa sp. H2 *cox1* gene, supporting the hypothesis of the existence of this exon in the common ancestor of all extant placozoans. The overall nucleotide sequence similarity of concatenated *cox1* exons (1.605 bp in total) between *Trichoplax adhaerens* and Placozoa sp. H2 is 99,7%. Three synonymous nucleotide substitutions are found in exon 2, while another two synonymous substitutions exist in exon 9 (see S1 Fig). The remaining seven exons are 100% identical at the nucleotide level between these two placozoan species. All observed substitutions are at the third position of the respective nucleotide triplet and none of these substitutions occur at splicing sites. The high sequence similarity as well as the identical *cox1* exon structure highlights the close relationship of the two placozoan species as already suggested by 16S rDNA phylogenetic analyses [24, 25]. Our RNA-seq data from Placozoa sp. H2 “Panama” furthermore confirm the unusual trans-splicing of *cox1* exons previously observed in *Trichoplax adhaerens*. Together, these similarities indicate that the *cox1* splicing mechanisms in both placozoans should be comparable (if not identical). However, as expected, the chronological order of *cox1* exon splicing events in Placozoa sp. H2 “Panama” cannot be reconstructed by short read RNA sequencing approaches, despite the long insert size paired-end library. It must be assumed that exons encoded nearby on the same strand are spliced together before trans-splicing of the three fragments occurs.

**Fig 2:**
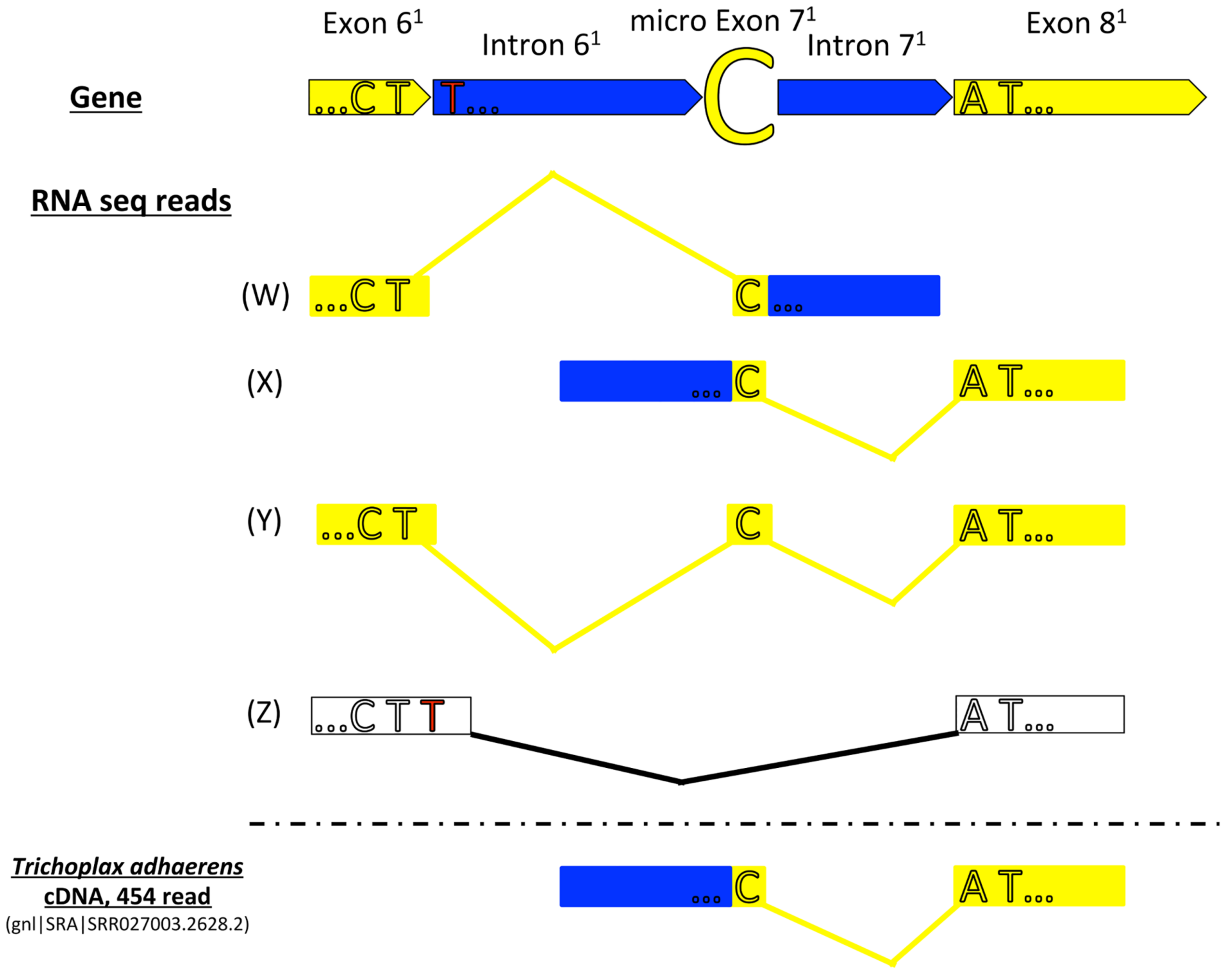
Schematic *cox1* transcript mapping. Shown are transcripts W, X and Y (assembled from multiple RNA-seq reads) and transcript Z (hypothetical transcript) mapped onto the partial *cox1* region of Placozoa sp. H2 “Panama”. Exon/intron color codes as in Fig 1. The superscript 1 indicates differences in the *cox1* annotation compared to Burger *et al*., 2009 (see text). Continuous reads/transcripts are indicated by yellow and black connector lines, respectively. The upper transcripts (W) and (X) represent intermediate splicing stages and transcripts from both directions overlap at the micro exon “C” (marked in yellow). Transcript (Y) represents the *cox1* mature mRNA sequence (in agreement with EST data from *Trichoplax adhaerens*). Transcript (Z) represents a hypothetical pre-mRNA-editing transcript (following Burger *et al*., 2009) which has not been found in our RNA-seq data. The putative mRNA editing site in transcript (Z) is indicated by a red “T”. The *Trichoplax adhaerens* cDNA read supporting the micro exon as well as the intron 7^1^ splice sites is illustrated at the bottom.

**Fig 3:**
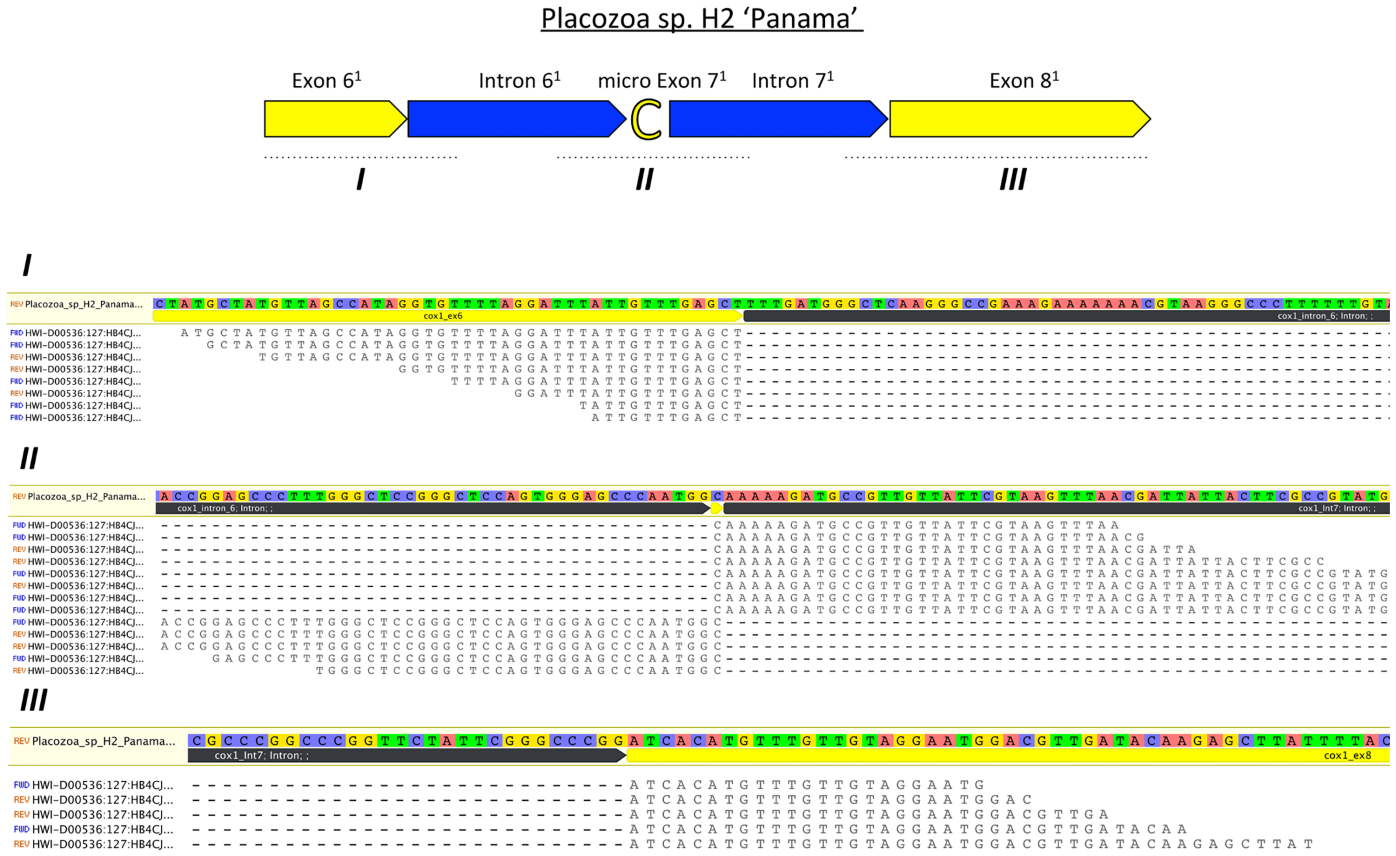
Mapping of RNA-seq reads on the partial Placozoa sp. H2 "Panama" *cox1* gene locus containing the micro exon. The *cox1* structure is given in the upper part. Exon/intron color codes are the same as in Fig 2. Mapping regions I, II and III are indicated by dotted lines and are enlarged below. Reads corresponding to transcript W (comprising exon 6^1^, micro exon 7^1^ and intron 7^1^) span region I and II while reads corresponding to transcript X (comprising intron 6^1^, micro exon 7^1^ and exon 8^1^) span region II and III, respectively. Continuous RNA-seq reads are connected by dashed lines (consequence of the applied gapped mapping procedure).

**Fig 4:**
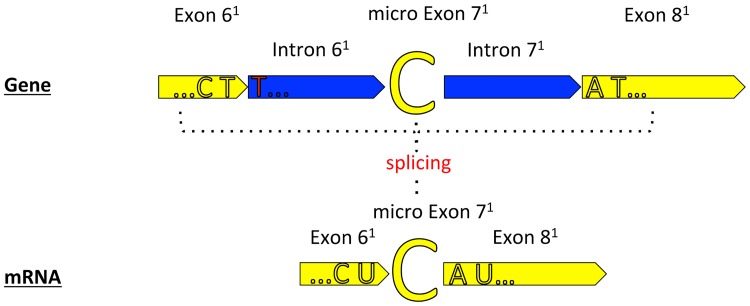
Placozoan *cox1* “micro exon” scenario. The scenario is based on Placozoa sp. H2 “Panama” RNA-seq data. Exon/intron color codes are the same as in Fig 2. Exon 6^1^ represents a truncated exon 6 (following Burger *et al*., 2009), which is indicated by the superscript 1. Subsequent exons/introns also differ in boundaries and/or numbering from the annotation by Burger *et al*., 2009 (likewise indicated by a superscript 1). The former intron 6 is now split into two introns (intron 6^1^ and 7^1^, respectively) flanking the newly identified micro exon 7^1^, which has been identified in this study. Splicing of exon 6^1^, micro exon 7^1^ and exon 8^1^ (formerly exon 7, Burger *et al*., 2009) leads to an in-frame coding sequence (CDS) with the intact CAT triplet coding for the functionally indispensable histidine at the respective position.

In order to screen for mRNA editing (U-to-C) in *cox1* we initially mapped RNA-seq reads to the concatenated Placozoa sp. H2 *cox1* coding sequence. We did not find a single unaltered (already spliced) *cox1* mRNA read (hypothetical transcript Z in Fig 2). There are two principle alternatives to explain this observation: (1) A short time window between splicing and mRNA editing may explain the absence of such a transcript due to insufficient coverage (despite an average 40x *cox1* coverage with RNA-seq reads); (2) The “C”, seen in all cases in the RNA-seq reads (transcript Y in Fig 2, 27x coverage) results from a previously missed exon. To test the second scenario we performed a gapped mapping of RNA-seq reads on the mitochondrial genome sequence to identify potential intermediate splicing stages (see Figs 2 and 3). Most of the mapped reads represented either unspliced mRNA (probably due to the polycistronic transcription of the mitochondrial genome) or already processed *cox1* mRNA (transcript Y, Fig 2). However, we identified 13 reads containing both, partial *cox1* exon and intron sequences, respectively, representing intermediate mRNA processing stages (Fig 3). In detail, we identified two overlapping read populations which were assembled into the two independent transcripts W and X, respectively (see Fig 2). Transcript W consists of the 3`end of exon 6^1^ (modified exon/intron numbering and/or boundaries compared to Burger *et al*., 2009 are indicated by the superscript 1), the micro exon 7^1^ and the 5`part of the intron 7^1^ while transcript X consists of the 3`end of intron 6^1^, the micro exon 7^1^ and the 5`part of exon 8^1^. Intronic sequence parts of transcripts W and X overlap in one base, i.e. the micro exon 7^1^ (see Figs 2 and 3). This indicates that the intron is spliced from both sites at this distinct position and that the micro exon 7^1^ (i.e. “C”) remains in the *cox1* mRNA after splicing. The truncation of the 3’ end of exon 6^1^ compared to the annotation by Burger *et al*. does not affect the group I intron splicing site (last base of exon 6^1^ is still a “T”), keeping the splicing capacity of the intron. The inclusion of the micro exon 7^1^ therefore does not only preserve the reading frame of the mRNA but also provides the crucial “C” for the “CAT” (histidine) codon by splicing (and without mRNA editing; see Fig 4). In order to exclude artifacts linked to the applied RNA sequencing method (i.e. short read sequencing) we confirmed the existence of transcript X (see Fig 2) in Placozoa sp. H2 “Panama” by long read Sanger sequencing (see Material and methods section). Furthermore, we also identified a transcript X cDNA read in the *Trichoplax adhaerens* 454-sequencing EST database (Fig 2). These data provide compelling evidence for the generation of the critical continuous “CAT”-triplet in the placozoan *cox1* mRNA by means of splicing.

**Fig 5:**
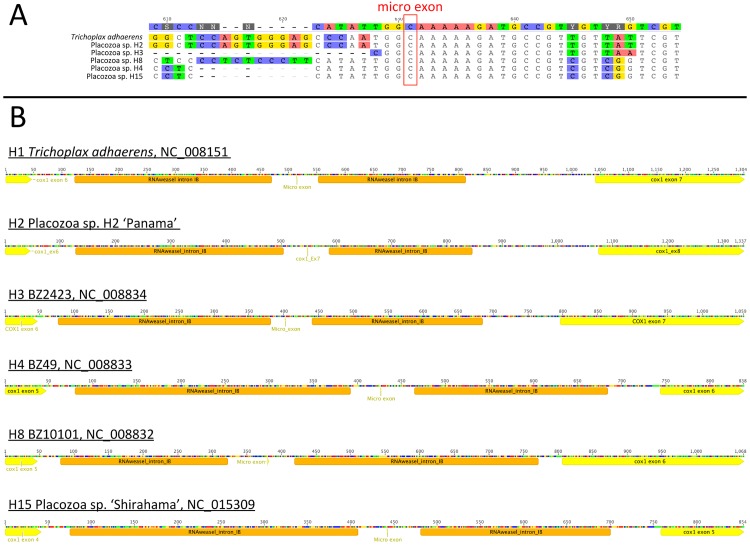
Conserved splicing sites and intron motifs in the placozoan *cox1* gene. A) Partial alignment of the placozoan *cox1* intron containing the predicted *cox1* micro exon. Although the overall pairwise sequence similarity of respective introns is low, the micro exon splicing motif can be found in all known placozoan mitochondrial genomes (for accession numbers see Material and methods). Numbers represent nucleotide position in the intron alignment. B) RNAweasel intron predictions annotated on partial placozoan *cox1* sequences. Conserved core group IB intron structures predicted by RNAweasel are illustrated in orange. In all known placozoan species the *cox1* micro exon is flanked by two independent introns, respectively.

We compared alignments of placozoan micro exon candidate regions and found support for the presence of the above scenario in placozoans in general. While the overall sequence similarity of flanking *cox1* introns is quite low, all sequenced placozoans posses a conserved putative splicing motif (GG/micro exon C/AA) in the respective *cox1* intron (Fig 5A) as well as a crucial “T” at the 3`end of the truncated *cox1* exon 6 (following the original *Trichoplax adhaerens* annotation, NC_008151).

**Fig 6:**
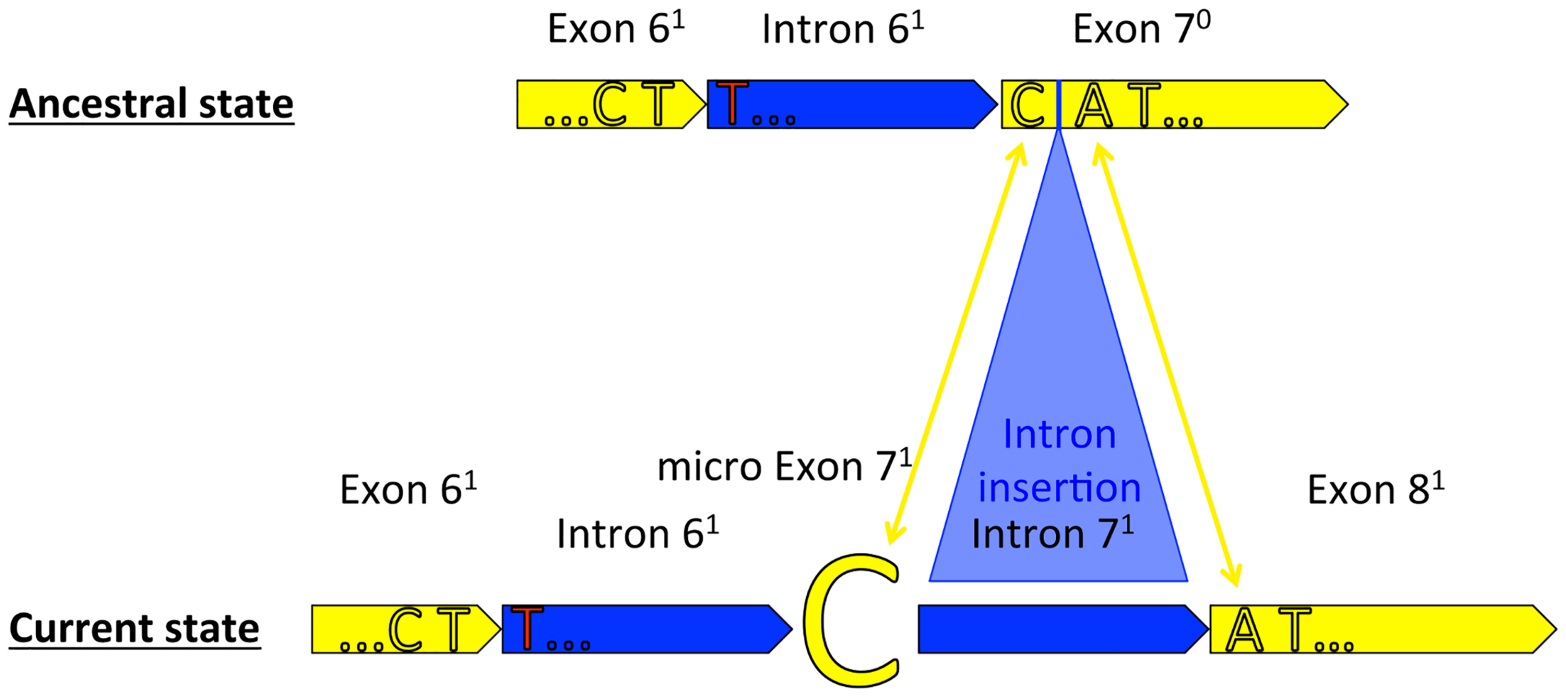
The evolutionary origin of the *cox1* micro exon in Placozoa. Exon/intron color codes are the same as in Fig 2. In the ancestral state the continuous CAT triplet was located in the hypothetical exon 7^0^ (comprising the later micro exon 7^1^ and exon 8^1^, this study). During course of evolution, the “C” of the conserved CAT triplet was isolated from the hypothetical exon 7^0^ by the insertion of a self-splicing intron 7^1^.

**Fig 7:**
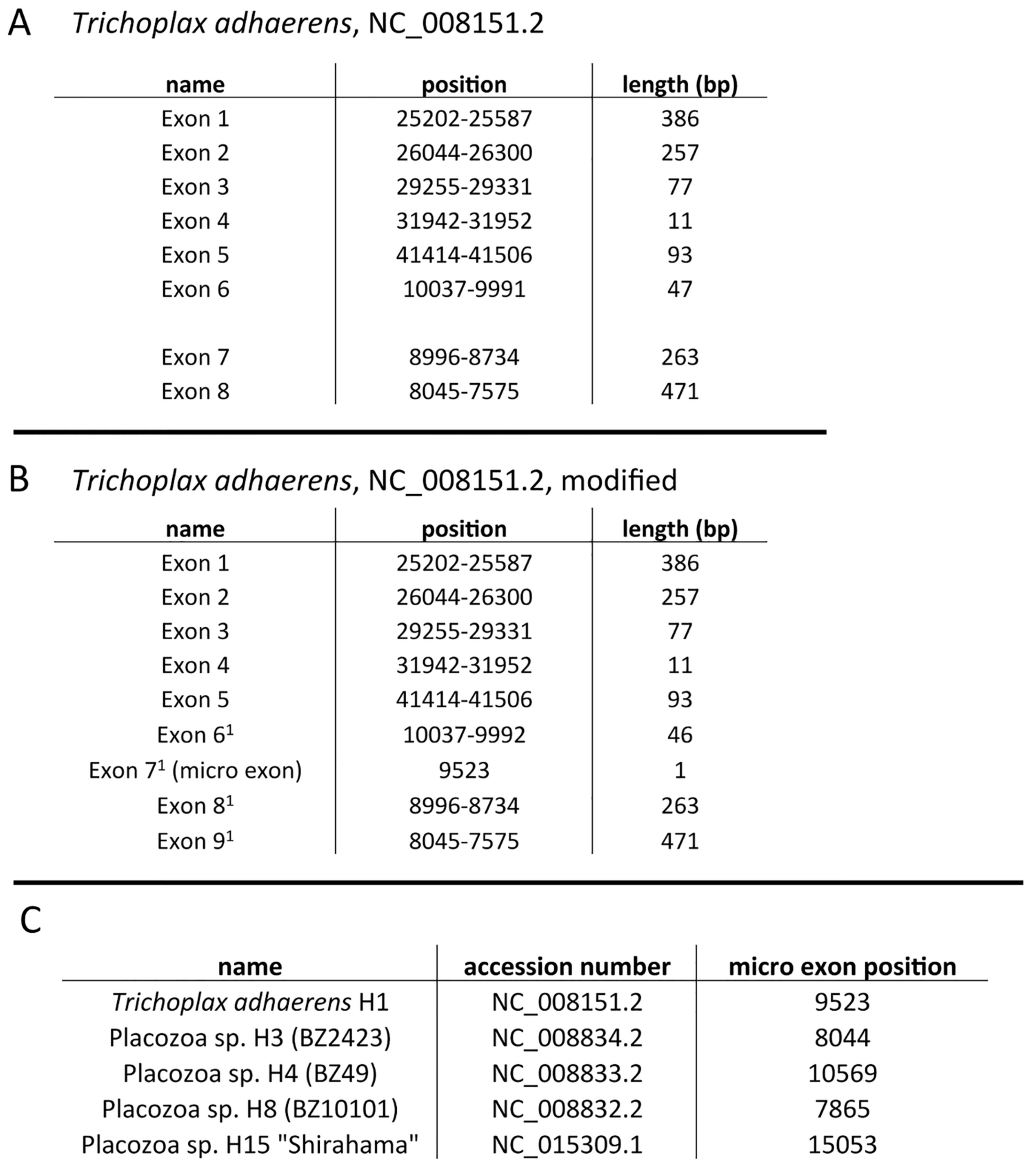
Comparison of placozoan *cox1* annotations. A) Positions and lengths of *cox1* exons in *Trichoplax adhaerens* according to Burger *et al*. 2009 (NC_008151.2). B) Differences in numbering, position and length of *cox1* exons in *Trichoplax adhaerens* considering the micro exon. C) Different positions of the *cox1* micro exon in all known placozoan mitochondrial genomes.

The single nucleotide micro exon in *cox1* seems to be a unique apomorphic character for placozoans and thus is not suited for evolutionary implications outside the Placozoa. Mitochondrial *cox1* introns are also found in other basal metazoans but previous analyses indicated independent intron gain events via horizontal or vertical transfer for instance within the Porifera [26]. Although there seem to be some general hot spots for intron insertions within the *cox1* gene in eukaryotes, comparison of intron positions between non-bilaterian animal phyla is still problematic and does not help to reconstruct the micro exon evolution in Placozoa. Albeit different evolutionary scenarios are possible we here discuss on the most parsimonious scenario of the micro exon “C” evolution and the related “CAT” triplet. The observed exon organization (with the last two nucleotides of the conserved “CAT” triplet located in exon 8^1^ in Placozoa sp. H2, Fig 4) suggests that the continuous “CAT” triplet was originally located in exon 7^0^ (Fig 6). The most parsimonious explanation for this scenario is that a self-splicing group IB intron 7^1^ integrated directly behind the “C” of the “CAT” triplet, i.e. after the first base of exon 7^0^ (see Fig 6). As a result the “C” (now micro exon 7^1^) was isolated from the rest of exon 7^0^ (which now becomes exon 8^1^). Further support for this hypothesis arrives from analyzing intron sequences in Placozoa sp. H2 using the RNAweasel tool [27]. The RNAweasel analysis revealed two individual group IB introns (see Fig 5B) flanking the micro exon 7^1^. Additional analyses of respective introns from other placozoans (Fig 5B, see also S1 Data) uncovered that all placozoans possess two distinct group IB introns (instead of one as suggested by Burger *et al*., 2009) flanking the single base pair micro exon (for instance, the micro exon “C” in the *Trichoplax adhaerens* mitochondrial genome (NC_008151.2) can be found at position 9,523 (see Fig 7)). This indicates that a single base pair has been isolated from the original exon by the insertion of a self-splicing intron probably before the radiation of placozoans. Although the isolation of a single nucleotide seems to be an unlikely event, it must be taken into account that placozoan mitochondrial genomes are characterized by large re-arrangements, intron gains and losses, and gene fragmentations, especially in *cox1* (see [12, 18] for details) and the 16S rDNA gene. The mechanisms are so far poorly understood and additional data from other placozoans are needed to reconstruct the evolution of placozoan mitochondrial genomes in detail.

Our analysis provides compelling evidence for the existence of a single base pair micro exon in *cox1*, which originates from the integration event of a self-splicing group IB intron. The RNA editing scenario as suggested by Burger *et al*. (2009) is not supported by our data. Also, any hypothetical evolutionary scenario that first shows a conserved “CAT” triplet (histidine), evolving into a “TAT” triplet (tyrosine) before an unknown mRNA editing mechanism switches the triplet back to a histidine could by no means be parsimonious. In sharp contrast, the micro exon scenario is supported by substantial empirical evidence and a well-known mechanism. Our results also highlight the importance of deep RNA sequencing for unraveling (unusual) transcription mechanisms in mitochondrial genomes in general.

## Material and methods

Long-term clonal cultures of Placozoa sp. H2 “Panama” have been maintained in the Schierwater lab as previously described [24, 28]. Total DNA and RNA isolations for Illumina sequencing were performed using standard phenol-chloroform-protocols [29]. Total DNA and unstranded total RNA paired-end sequencing was conducted at the Yale Genome Center on an Illumina HiSeq 2500 and Illumina GAII, respectively. The complete mitochondrial genome of Placozoa sp. H2 “Panama” was reconstructed using an iterative mapping and consensus call approach as implemented in Geneious v. 8.x [30] starting with a published 16S rDNA sequence as ‘seed’ (see e.g. [31–33]). The mitochondrial genomic *cox1* region of Placozoa sp. H2 “Panama” was identified and annotated using BLASTx [34]. RNA-seq reads (76bp read length) were mapped to the target mitochondrial genome region as well as to the complete *cox1* mRNA sequence using high stringency settings and gapped mapping approaches (implemented in Geneious v. 8.x). For validation of transcript X (see Fig 2) by means of PCR, total RNA from 100 individuals of Placozoa sp. H2 “Panama”was isolated using standard phenol-chloroform protocols. Digestion of DNA was conducted using DNase I (Thermo Scientific) and purity of RNA was checked on an agarose gel (SeaKem LE agarose, Lonza). cDNA synthesis was performed using Superscript II reverse transcriptase (Invitrogen) using random hexamer primer following manufacturer’s recommendations. All PCR experiments were performed using the MyTaq system (Bioline) on an Eppendorf Mastercycler. In detail, in a first PCR step mRNA transcripts spanning the region from *cox1* exon 6^1^ to *cox1* exon 8^1^ were enriched using primer H2_COX1_6_FW (5´-tgttagccataggtgttttagga-3´) and H2_COX_1_8_RV (5`-tgcgaccactaccactaaca-3`). In a second nested PCR step (using target enriched template from the first PCR step) transcript X (spanning intron 6^1^, micro exon 7^1^ and exon 8^1^) was amplified using primer H2_INTRON_6_FW (5`-gctcaagggccgaaagaaaa-3`) and H2_COX_1_8_RV (5`-tgcgaccactaccactaaca-3`). The length of PCR products was checked on an agarose gel and bands of expected length were cut out and purified using standard gel extraction protocols. Target PCR products were cloned in a pGEM-T Vector (Promega) and transformed in *E*. *coli* Top10 cells following the manufacturer’s protocol. Candidate bacterial colonies containing the expected vector insert were identified via blue-white screening on agar plates (containing ampicillin + X-gal). Colonies were screened by colony PCR using standard vector specific primers T7 and SP6. PCR products were checked on an agarose gel and purified using standard PCR purification protocols. Sequencing of PCR products was conducted at Macrogen Europe. For long-term storage a glycerol stock of a bacterial colony containing the cloned transcript X (see Fig 2) has been prepared and is available upon request. Mitochondrial genome sequences from previously published placozoan mitochondrial genomes were downloaded from NCBI (*Trichoplax adhaerens* H1 (NC_008151), Placozoa sp. H3 (NC_008834), Placozoa sp. H8 (NC_008832), Placozoa sp. H4 (NC_008833) and Placozoa sp. H15 (NC_015309)). *Cox1* intron structures were reanalyzed using RNAweasel (http://megasun.bch.umontreal.ca/cgi-bin/RNAweasel/RNAweaselInterface.pl) [27]. Alignments of *cox1* mRNA and intron sequences were performed using MAFFT v.7.017 [35] (implemented in Geneious v. 8.x).

## Supporting information

S1 Fig Nucleotide alignment of concatenated *Trichoplax adhaerens* (H1) and Placozoa sp. H2 “Panama” *cox1* exons. Exons are shown in yellow with arrowheads marking ends. Single nucleotide substitutions in exon 2 and exon 9 are highlighted. Amino acid sequences (code 4; i.e. mold, protozoan and coelenterate mitochondrial code) are given below the nucleotide sequences.
(TIF)

S1 Data Output of RNAweasel analyses of placozoan mitochondrial introns containing the *cox1* micro exon. (TXT)
