# Supplementary figures and images for: Deep RNA sequencing reveals the smallest known mitochondrial micro exon in animals: The placozoan cox1 single base pair exon
Source: PLoS One. 2017 May 18;12(5):e0177959. doi: 10.1371/journal.pone.0177959 (PMC5436844; doi:10.1371/journal.pone.0177959)

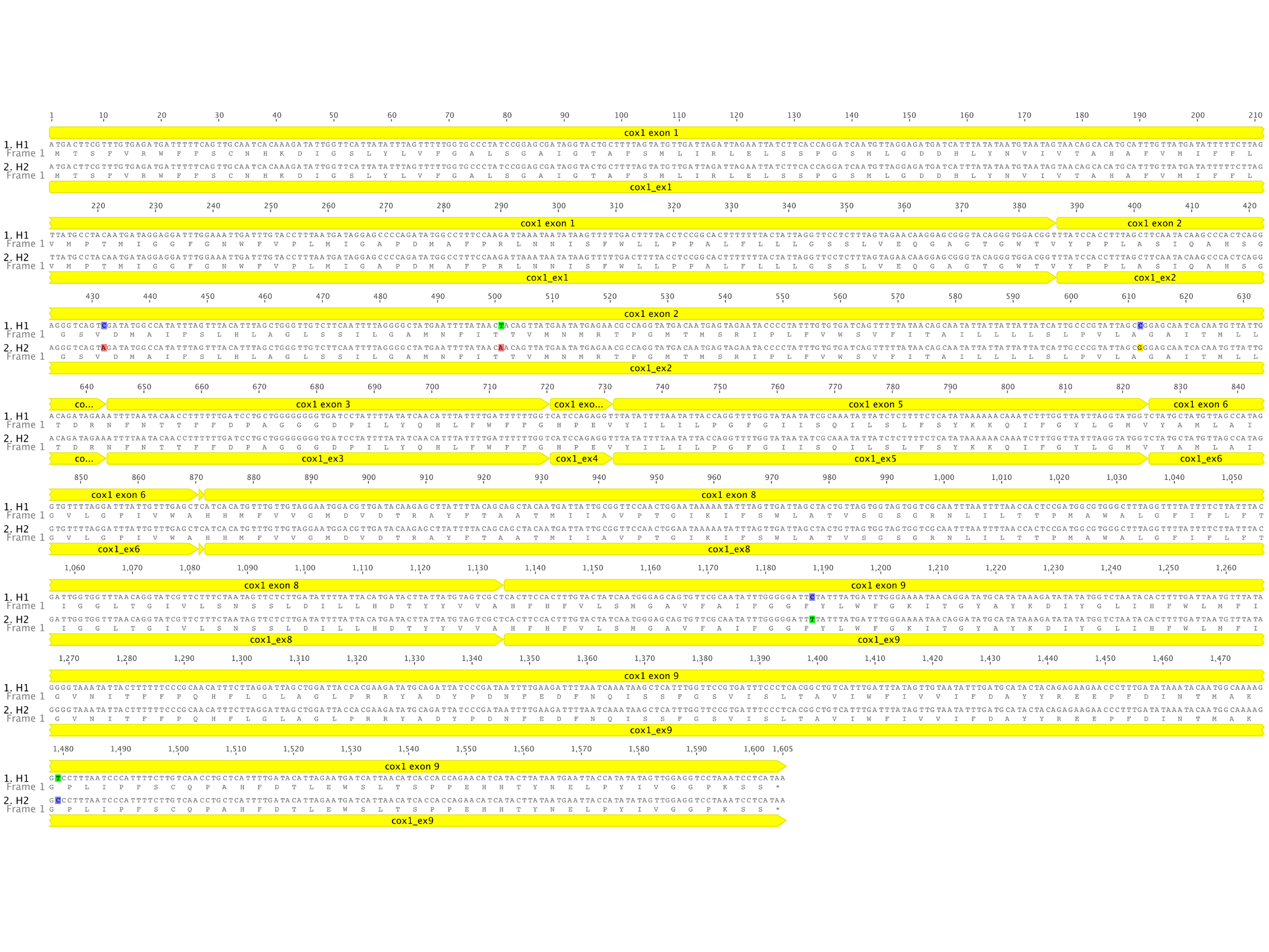

Supplement: S1 Fig — Exons are shown in yellow with arrowheads marking ends. Single nucleotide substitutions in exon 2 and exon 9 are highlighted. Amino acid sequences (code 4; i.e. mold, protozoan and coelenterate mitochondrial code) are given below the nucleotide sequences. (TIF) [file pone.0177959.s001.tif]
